# Psychometric evaluation of the perceived access to health care questionnaire

**DOI:** 10.1186/s12913-021-06655-2

**Published:** 2021-07-02

**Authors:** Sara-Sadat Hoseini-Esfidarjani, Reza Negarandeh, Farzaneh Delavar, Leila Janani

**Affiliations:** 1 Nursing and Midwifery Care Research Center, Tehran University of Medical Sciences, Nosrat St., Tohid Sq, Tehran, 1419733171 Iran; 2 Department of Community Health & Geriatric Nursing, School of Nursing and Midwifery, Tehran University of Medical Sciences, Tehran, Iran; 3 Department of Biostatistics, School of Public Health, Iran University of Medical Sciences, Shahid Hemmat Highway, Tehran, 1449614535 Iran

**Keywords:** Validation study, Perception, Health care services, Health care services access, Factor analysis

## Abstract

**Background and objective:**

Access to health care is a universal concern. Therefore, this study was conducted to develop a questionnaire to assess the Perceived Access to Health care based on Penchansky and Thomas’s definition of access and the assessment of its psychometric properties.

**Method:**

The initial questionnaire contains 31 items developed based on a deductive approach with an extensive review of the related literature. Content validity, face validity, construct validity, internal consistency, and instrument reliability were further examined. Data analysis was conducted using SPSS software version 24, R software version 4, and lavaan package.

**Results:**

The initial questionnaire was examined using qualitative content validity, and the necessary modifications were applied to each item. The content validity ratio (CVR) was approved in 30 items with a value greater than 0.78, and one item with a CVR value lower than 0.78 was removed. In the case of the content validity index (CVI), 29 items were approved with a CVI value of greater than 0.79, and one item with a CVI value between 0.70 and 0.79 was revised. In qualitative face validity, all items were approved by a panel of experts and the participants. All 30 items with an impact score index higher than 1.5 were approved for the next steps. The confirmatory factor analysis results showed that the six-factor model of access to health care has an appropriate fit. Cronbach’s alpha coefficient for the questionnaire was calculated 0.86. The value of Cronbach’s alpha for the dimensions of availability, accessibility, affordability, accommodation, acceptability, and awareness were 0.61, 0.76, 0.66, 0.60, 0.80, and 0.76, respectively. The Intraclass Correlation Index (ICC) value for reliability (test-retest) of the whole instrument was calculated 0.94 using the two-way mixed absolute agreement method.

**Conclusion:**

The success of health programs depends on eliminating barriers to access to provided health care services. One of the most critical barriers to understanding access is a perception of limited access. This questionnaire might be used further to understand perceived health care access in different global contexts.

**Supplementary Information:**

The online version contains supplementary material available at 10.1186/s12913-021-06655-2.

## Introduction

Healthcare access is a complex and global issue recognized as a fundamental human right [1]. Access to health care services plays a crucial role in the healthcare system’s efficiency worldwide [2]. Hence, access is considered one of the main public policy issues in setting priorities or evaluating the healthcare system’s performance [3]. Equality in health requires access to high-quality health care services for all individuals and groups of people. Therefore, facilitating and maintaining individuals’ access to essential health care services is one of the most important strategies to pursue to achieve social justice in each country’s healthcare system [4].

A minimum of 400 million people worldwide is currently deprived of access to basic health care services. In addition, one out of every five people in the world lives in areas that are experiencing humanitarian crises [5]. Access problems, especially in low-income countries, actually threaten the achievement of the goals that the United Nations set in the Millennium Development Goals [6].

Evidence suggests that horizontal justice or equal access to equal needs in developed countries is also different for many care types [7]. Differences in access to health insurance between different races and individuals cause the death of more than 100,000 people each year [8]. Minorities often do not have access to health care services in a timely manner. The barriers to access to health care services have caused most minorities to experience delayed access to health care services or be ignored altogether. Such conditions lead to an increase in the cost of health care services, so that the progress in health care over the past 30 years has not shown a significant improvement in the rate of mortality among the minorities [9]. Shortage of services, distance from services, and inappropriate distribution of health care providers have limited access to health care services for rural residents [10]. Some Europeans still feel they are unable to access healthcare; in some European countries such as Bulgaria, Croatia, Latvia, Poland, Romania, and Sweden, reports suggest that more than 10% of the population do not access health care services [7].

Historically, the concept of health care services access has had different definitions that correspond to each health system’s structural values. Most of the access models and definitions are based on market logic and meeting the needs; however, only very few models and definitions consider health as a basic human right [11].

Since the early 1970s, there has been extensive debate about the concept of health care access that still remains confusing [12]. Some researchers have used this term to denote the use of health care services. In contrast, other researchers use this term to convey a broad and multidimensional concept that describes the health system’s characteristics [3]. Through analyzing this concept, Penchansky and Thomas (1981) have defined five dimensions for access, including 1) availability, 2) accessibility, 3) affordability, 4) accommodation, and 5) acceptability [13].

The first two dimensions are spatial in nature. Availability refers to the total number of services that the consumer can choose to receive. The accessibility dimension refers to the commuting time and the distance between the consumer’s residence and the service center. The next three dimensions are non-spatial and belong to cost, service quality, and cultural factors [14]. For further explanation, it can be said that the availability dimension refers to the physical availability of health care resources with sufficient capacity to provide services [2]. Finally, the accessibility dimension refers to the location of health care delivery and the patient’s residence, which includes concepts such as commuting time and distance [13].

The financial dimension shows the economic capacity of people to use the appropriate services [2]; in other words, this dimension indicates the relationship between the costs of health care providers and health insurance, the patients’ ability to pay the prices, and their income [13]. The accommodation dimension is related to how the services are organized [15] and the provision of services based on the patients’ needs and expectations. Finally, the acceptability dimension is related to the patients’ and health care providers’ personality traits and the impact of these traits on their attitudes and behaviors [13].

In 2015, Saurman added another dimension, which was called “awareness”, to the model proposed by Penchansky and Thomas. By definition, awareness is the identification of the need for health care services. It is essential to know who the target of the services is, what they do if they are available, where and how they are used, and why they are used. Awareness also includes health literacy as another component of this dimension [1].

It can be said that access and use are not synonymous [16]. Access refers to the ability to receive the appropriate care from a proper healthcare provider, at the right time and place, depending on the context [1]. The decision to use available health care services depends on individuals’ perception of the services [17]. In general, recognizing the perception of access to care for those in need is the first important step in reducing barriers to access [18]. Consumers’ perception of access to health care services is crucial [10] because the decision to use available health care services depends on individuals’ perception of the service and its reasonable costs [17]. Individuals’ perceptions and judgments are often created by assessing the factors that make their traditions and culture important. These perceptions derived from their knowledge, values, ​​and attitudes affect the place, time, and even whether patients should seek health care services or not [10]. To achieve public health, individuals’ perception of health care services is essential for all the stakeholders in order to be able to provide successful interventions [17]. Perception enables judgment, and perception of access to health care services is, in fact, an alternative to demonstrating access to health care services that examines access management in a more straightforward way [11].

While there are specific indicators to measure different access dimensions separately, there is no extended indicator available. For example, health care services’ availability has traditionally used indicators per capita of medical staff and resources such as hospital beds and medical technology units. Although these indicators show some of the available resources, they do not demonstrate anything about the quality of these resources or whether people should use them or not. Newer indicators of service availability such as distance, commuting time and cost, or public transportation to the nearest medical facilities can pose other methodological problems. These data underestimate the patients’ actual communication because the closest service providers are not always the individuals’ choice. In addition, the characteristics of each health system may widely vary across countries; given the development of disease management programs in many countries, measuring access to health care services will become increasingly difficult [19]. The literature review showed that many studies did not have appropriate and standard instruments to measure individuals’ perception of healthcare services access [7, 17, 20].

In Quinn’s study, the dimensions of access in existing questionnaires have been investigated. From this study, it can be understood that each of the existing questionnaires focused on a specific dimension of access, and the available questionnaires did not examine all dimensions comprehensively and simultaneously [21]. Also, a study was observed on the development and validation of instruments for the perception of access to health care services [3], which did not cover all the dimensions of the present study; Besides, the present study applied a more robust method to analyze experimental data. Given the importance of individuals’ perception of their access to health care services and the need to develop standard instruments to measure it, this study was conducted to develop and evaluate psychometric properties of the questionnaire to assess perceived access to health care services among the adults.

## Method

### Designing of the questionnaire

The initial version of the Perception of Access to Health care Services Questionnaire was developed based on the deductive method with a wide range of contents, definitions, and features of the concept of access as well as the perception of access and related questionnaires. The initial questionnaire was developed according to the five dimensions, which were introduced in Penchansky and Thomas’ model of access (1981), and the sixth dimension, which was introduced in Saurman’s (2015) study [1]. Thus, the proposed questionnaire includes six dimensions: 1) availability, 2) accessibility, 3) affordability, 4) accommodation, 5) acceptability, and 6) awareness. In addition, the psychometric evaluation was conducted on the instrument’s initial version with 31 items on a 5-point Likert scale (strongly agree to strongly disagree).

### Content validity

The content of the questionnaire was examined in both quantitative and qualitative ways. Ten expert panel members were selected through the convenience method for the qualitative assessment of content validity. The opinion provided by the panel of experts in the field of access to health services, research, and psychometrics was obtained in terms of grammar, use of appropriate items and phrases, proper location of questionnaire items, and scoring procedure. Moreover, the questionnaire was sent to 15 experts for the quantitative analysis of the content validity, and the content validity ratio (CVR) and content validity index (CVI) were determined. In order to calculate CVR and after explaining the objectives of the questionnaire and providing the operational definitions related to the content of the questions, the experts were asked to rate each question based on a three-point Likert scale, “The item is necessary”, “The item is useful but unnecessary”, and “The item is unnecessary”. Then, the content validity ratio (CVR) was calculated using the formula.^1^ In examining the Content Validity Index (CVI), the experts were asked to determine the degree of relatedness based on a four-point scale: 1) not relevant, 2) somewhat relevant, 3) quite relevant, 4) highly relevant. The number of experts who chose options 3 and 4 was divided by the total number of experts afterward.

### Face validity

In order to determine the face validity using the quantitative method, the items in the developed instrument were measured based on a five-point Likert scale quite important [5], important [4], moderately important [3], slightly important [2], and unimportant [1]. In this regard, the proposed questionnaire was provided to a panel of 20 experts. Thus, the ratio of participants who had chosen 4 and 5 for each item (frequency), as well as the sum of scores assigned to each item and the average score of each item (importance) were determined. Then, using the formula,^2^ the Impact score of each item was calculated. An Impact score equal to or greater than 1.5 was considered acceptable for each item. On the other hand, based on purposeful sampling, ten members of the panel of experts (written and email) and 30 members of the target group (face to face) were selected to conduct qualitative face validity. They were asked to comment on the level of item difficulty, comprehensibility of the phrases and the words, the appropriateness and relatedness of the items, as well as the ambiguity and misinterpretation of the words and phrases.

### Construct validity

The construct validity determines the extent to which observational variables (items) can explain the study’s main concept (the concept of perception of access to health care services). In the construct validity stage, the statistical population included patients referred to healthcare centers in the south of Tehran who were selected based on multi-stage sampling. Therefore, in the beginning, two areas were randomly selected from all the areas (5 areas) covered by the South Tehran Health Center. Then, two health centers were randomly selected from the health centers of these two areas. After that, the required samples were selected consecutively from those referred to these two health care centers. Inclusion criteria for the present study include the ability to speak in Persian, no apparent cognitive impairment, no history of specific diseases (dialysis, hemophilia, multiple sclerosis, and renal transplantation), citizenship and residence of Iran, recipients of services from health centers, and the age range between 20 and 60 years. According to the study of Myers et al., 300 people were considered as the sample size at this stage [22]. The objectives of the research were explained to all the participants before collecting data. They were then reassured about the confidentiality of the information, and informed consent was obtained.

The questionnaires were also completed through interviews with the participants from November 2018 to June 2019. The construct validity was determined using confirmatory factor analysis. Fit indices were used in the confirmatory factor analysis with experimental data. Model fit was considered good if the Tucker Lewis Index (TLI) [23], Normed-Fit Index (NFI), Non-Normed-Fit Index (NNFI), Goodness of Fit (GFI) were greater than or equal to 0.95. Comparative Fit Index (CFI) and Adjusted Goodness of Fit (AGFI) were greater than or equal to 0.90 [24]. Root mean square error of approximation (RMSEA) was less than 0.05. The ratio of χ2 to degrees of freedom (χ2/df) was below 2 [23].

### Reliability

Reliability refers to the degree to which individuals maintain their position in a sample of repetitive dimensions. To assess it, the Intraclass Correlation Index (ICC) value was measured using the two-week test-retest approach for a group of 10 people from the Southern Health Centers [25]. In this study, the minimum ICC rate was considered 0.75 [26].

Internal consistency indicates whether the items in an instrument are conceptually compatible or not. Internal consistency was assessed using Cronbach’s alpha. Besides, the internal correlation of the final questionnaire was examined, with 300 patients referred to the selected health centers. In this study, Cronbach’s alpha of greater than 0.6 was considered acceptable [27, 28].

### Data analysis

Mean (standard deviation) was used to describe quantitative variables, and frequency report (percentage) was used to describe categorical variables. These analyzes were performed in SPSS software version 24. Also, R software version 4 and lavaan package were used for confirmatory factor analysis. Substitution of the variable’s mean for the missing data points on that variable was used for the missing data. In addition, fit indices were used in the confirmatory factor analysis with experimental data.

### Findings

In qualitative content analysis, the questionnaire was reviewed, and the necessary corrections were applied to each item based on the opinions obtained from the panel of experts. It should also be noted that only 9 of the 15 experts invited to the panel responded. Therefore, according to the numbers provided by the Lawshe table, the items with a CVR coefficient of greater than 0.78 were considered acceptable, and their validity was confirmed. Accordingly, 30 items with a CVR value of greater than 0.78 were confirmed in the present study. However, one item with a CVR value of lower than 0.78 was removed. Also, the content validity index (CVI) was evaluated for 31 items. In the case of CVI, the items with a score higher than 0.79 were considered appropriate, the items with a score between 0.70 and 0.79 were considered as the items with the need to be revised, and the items with a score equal to and lower than 0.70 were considered as unacceptable items and were then removed. Accordingly, 29 items with a content validity index value of greater than 0.79 were confirmed. A single item with a score of between 0.70 and 0.79 was revised. The item “When providing education, the underlying factors, as well as my ability to understand health information, are taken into account.” in the dimension of “awareness” was changed to “My living conditions are taken into accounts, such as marital status, ability to pay, and cultural differences”. The item “I recognize the need for specialized and sub-specialized services” in the dimension of Affordability with a score of lower than 0.70 was removed. In fact, the same item was removed in both CVI and CVR.

As a result, the remaining 30 (see technical appendix in Additional file 1) items in the content validity analysis were examined for face validity. Findings from the qualitative face validity showed that the panel of experts and participants approved the level of difficulty, the degree of appropriateness and ambiguity of the scale, and the 5-point Likert scale. In the quantitative face validity analysis, the item effect size was calculated, and all items were retained for the next steps since the obtained score for each of the 30 items was higher than 1.5.

Thus, the final questionnaire with 30 items and six constructs was completed through interviews with 300 individuals referred to the research setting. Finally, the data for 276 individuals were analyzed because some data within 24 questionnaires were incomplete. The participants aged between 20 and 60 years with an average of 37.8 years and a standard deviation of 10.27 years.

Table 1 shows the distribution of the participants’ demographic characteristics (sex, level of education, place of employment, insurance status, and supplementary insurance).

**Table 1:** Demographic characteristics of the individuals referred to healthcare centers (*n* = 276)

Demographic characteristics		Frequency	
		Number	Percentage
Sex	Female	212	76.9
	Male	60	21.7
	Unanswered	4	1.4
Educational level	Illiterate	11	4
	Elementary or Middle school	79	28.6
	High-school diploma	97	35.2
	Graduated	58	30.8
	Unanswered	4	1.4
Occupation	Employed	95	34.4
	Unemployed	179	64.9
	Unanswered	2	0.7
Insurance status	Supported	224	81.2
	Unsupported	46	16.7
	Unanswered	6	2.1
Complementary insurance status	Supported	57	20.7
	Unsupported	213	77.2
	Unanswered	6	2.1

**Fig. 1:**
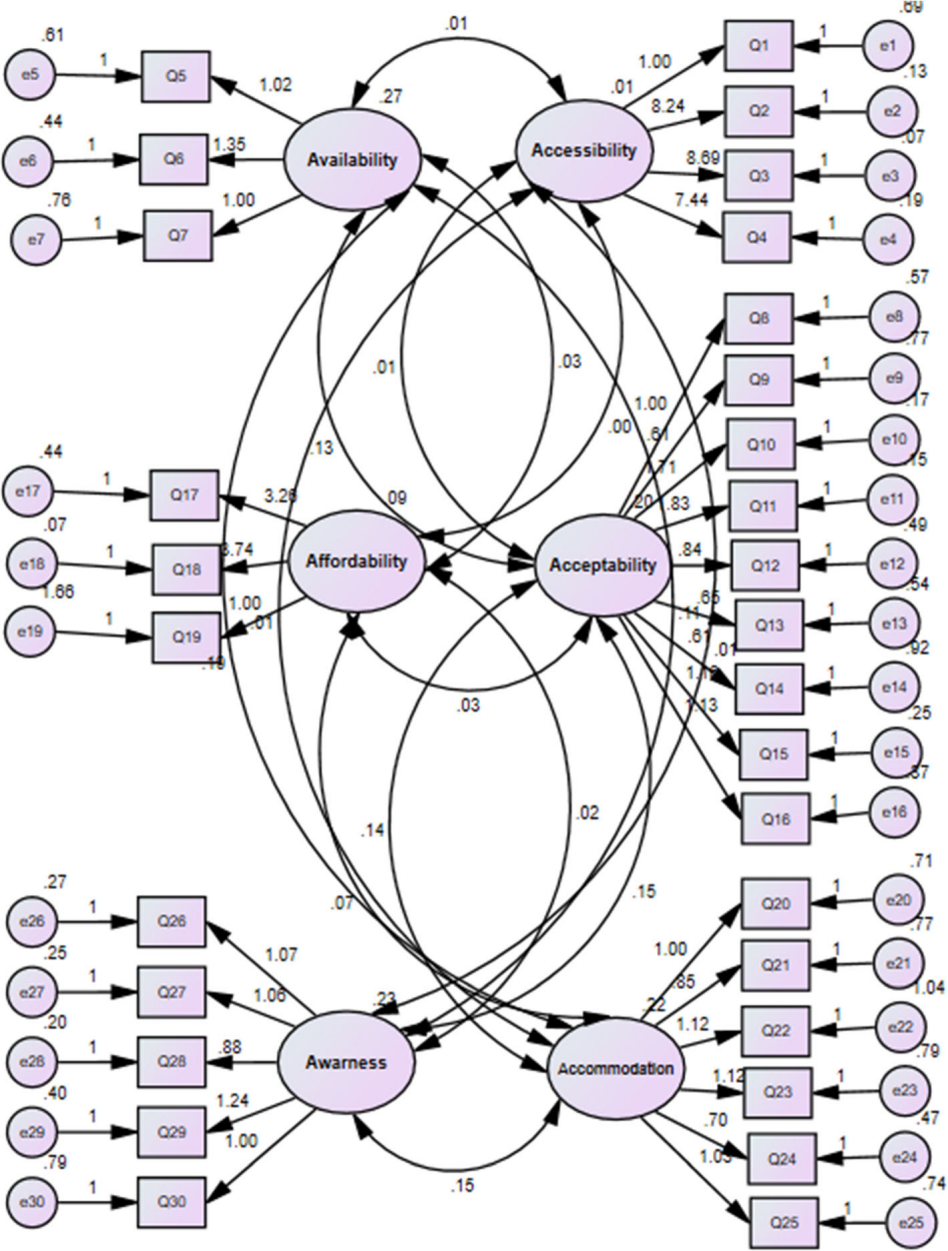
Structural Equation Modeling

The confirmatory factor analysis results showed that the six-factor model of perception of access to health care services in patients referred to health centers provides a good fit. GFI, AGFI, TLI, NNFI, NFI, CFI, RMSEA, and χ^2^/df were calculated; the indicators confirm the model fit and acceptable values given in Table 2. Figure 1 shows the factor loading of latent factors.

**Table 2:** Fit indices for confirmatory factor analysis model (*n* = 276)

Model fit indices	χ2/df	RMSEA	GFI	AGFI	NNFI	TLI	NFI	CFI
Confirmatory factor analysis	1.147	0.024	0.938	0.926	0.981	0.981	0.884	0.983
Thresholds for acceptable fit	< 5	< 0.08	> 0.8	> 0.85	> 0.80	> 0.80	> 0.80	> 0.85
Thresholds for good fit	< 2	< 0.05	> 0.95	> 0.90	> 0.95	> 0.95	> 0.95	> 0.90

Table 3 shows the internal consistency of the Perception of Access to Health care services Questionnaire using Cronbach’s alpha. It is reported that the Cronbach’s alpha for the subscales of the Perception of Access to Health care services questionnaire was calculated between 0.60 and 0.80. While the value of Cronbach’s alphafor three dimensions was (0.6–0.7) indicates an acceptable level of reliability [28], the rest have good, with The value of Cronbach’s alpha greater than 0.7. However, the obtained Cronbach’s alpha for the whole questionnaire was 0.86. Besides, the questionnaire’s obtained ICC value was 0.94 (CI95%: 0.78, 0.98), which was calculated using the two-way mixed absolute agreement method.

**Table 3:** The Cronbach’s alpha coefficient for the Perception of Access to Health care services Questionnaire

Dimensions	The number of items	Reliability (Cronbach’s alpha coefficient)
Availability	3	0.61
Accessibility	4	0.76
Affordability	3	0.66
Accommodation	6	0.60
Acceptability	9	0.80
Awareness	5	0.76
Perception of Access to Health care services	30	0.86

## Discussion

Access to health care is an important concept in public health [29]. Access to health care can be defined in many ways. Some definitions address whether people actually use such services or not and define their use of services as access. Other definitions focus on health insurance coverage or eligibility to receive health care in the event of illness. Some other definitions highlight the possibility that someone can access health care services if needed [30]. In general, access to health care services is traditionally measured using population-level parameters such as the availability, distribution, and proximity of health facilities to the population. These approaches do not consider important factors such as individuals’ attitudes, perceptions, expectations, experiences, and socio-cultural factors such as norms and belief systems that affect people. In contrast, these factors can enable or hinder access to health care [29].

Evaluation of the success of executive programs in access to health care services requires a valid measure of the perception of access to health care services; the questionnaire that was developed and underwent psychometric evaluation in this study could help achieve such measurement.

Some studies have been conducted on the perception of access to health care services [7, 17, 31, 32]. However, none of them used a valid and well-known instrument. These studies have only examined one or more access dimensions that could not cover all the present study areas. Pyne et al. (2020) developed an instrument to measure the perception of access to mental health care services; nonetheless, this study did not aim to validate the instrument [33]. Zandom et al. (2017) developed a scale to measure perceptions of access to health care services among the disabled and their non-disabled counterparts in Nigeria. This instrument included 25 items and was developed based on the theories proposed by Levesque et al. (2013), Peters et al. (2008), Obrist et al. (2007), and Penchasky & Thomas (1981) [3].

It can be claimed that the questionnaire which was developed in the present study is distinct because it is based on the modified Penchansky and Thomas’s Theory of Access [1]. Their model provides a classified definition of access and summarizes a set of specific dimensions that describe the fit between the health care system and the general population. All dimensions of access have equal values in this theory, just like chain knots [34].

Accordingly, the questionnaire for perception of access to health care services was developed in six subscales and 31 items in the present study: 1) availability which refers to the adequacy of supply and examines the available services and resources to meet the potential and actual needs of the consumers and communities; 2) accessibility which evaluates the available service in accordance with the reasonable proximity to the consumer in terms of time and distance; 3) affordability which refers to the direct costs to the health care providers and consumers; 4) accommodation which refers to the adequacy of classification of services in order to be accepted and used by the consumers; 5) acceptability which highlights the provider and consumers’ attitudes about the characteristics of the service as well as their social or cultural concerns; and 6) awareness represents the concept that services can help maintain awareness through effective communication and information strategies with the target users (physicians, patients, and the general public), including attention to health context and literacy [1].

In both the quantitative and qualitative approaches to content validity, only one item was removed, while all the items were retained in the analysis of face validity. Thus, the questionnaire for the perception of access to health care services was observed for construct validity with six subscales and 30 items. In addition, confirmatory factor analysis was used to evaluate the construct validity of the Perceptions of Access to Health care services. The desired values ​​of fit indicators in confirmatory factor analysis confirmed the construct validity of the Questionnaire and indicated the proper classification of the questions. A good questionnaire is generally supported by a reasonable theoretical basis so that the items can operationally define the targeted questionnaire [35, 36].

For this reason, confirmatory factor analysis (CFA) has been used in the present study. Moreover, CFA is used when there is a strong model assumption. CFA aims to investigate the existence of a previously confirmed construct with a new dataset [37] However, in the study of Zandom et al.; The construct validity showed that the items of the questionnaire were related to the six-dimensional hypothetical structure based on models of access to health services [3].

Cronbach’s alpha was calculated to estimate the total instrument’s internal consistency and its different dimensions; the obtained Cronbach’s alpha for the entire questionnaire was 0.86 and for the dimensions were between 0.6 and 0.8. Besides, there are several reports of acceptable alpha values ​​ranging from 0.70 to 0.95. A seminal study by Tavakol and Dennick noted that if Cronbach’s alpha is too large, it may indicate redundant items that raise similar questions with only different phrases. Therefore, the maximum alpha value is recommended to be 0.90 [38]. Although the whole instrument’s internal consistency and its three dimensions (awareness, acceptability, and acceptability) were good, the other three dimensions (availability, affordability, and accommodation) of the questionnaire had acceptable Cronbach’s alpha. Tavakol and Dennick (2011) wrote that “A low value of alpha could be due to a low number of questions, poor interrelatedness between items or heterogeneous constructs” (p.54) [38].

In this study, the dimensions of availability and affordability in the perceived access to health care questionnaire had only three items that could explain the low Cronbach’s alpha. Although the dimension of accommodation has six items, Cronbach’s alpha in this dimension was also low, which could be due to the cultural differences of the study participants. Jih-Yuan Chen’s study suggests that low Cronbach’s alpha levels may be due to cultural bias [39]. Tehran (research setting) embraces people with different cultures. It may be justified that understanding people with different cultural backgrounds has caused them to answer the following questions in different ways. Also, there are broader concepts in this dimension than other dimensions that increase the possibility of heterogeneous items. This questionnaire is designed based on the modified Penchasky & Thomas model. It seems that the development of the model in the future can provide more clarity in the definition of its dimensions and help revise the questionnaire items accordingly. Nevertheless, the dimensions with low Cronbach’s alpha need to be further examined in future studies.

In addition, the Intra-Class Correlation Index (ICC) was calculated to determine the reliability of the instrument using the test-retest approach. ICC is a reliable indicator that reflects both the degree of correlation and the compatibility between the measurements; the obtained ICC value in the present study was equal to 0.94. According to Koo and Li, this rate indicates excellent reliability of the questionnaire [26].

It can be concluded that the “Perceived Access to Health care Questionnaire” was developed with 30 items; it has acceptable content validity, face validity, construct validity, and reliability. Therefore, this scale can be used in future studies to measure perceived access to health care services.

In some cases, perceptions about access to health services can be a barrier to using health services; we can identify these mental barriers and evaluate interventions’ effectiveness by measuring them. The removal of such mental barriers to greater access to the services helps to achieve equity in access.

A limitation of this study is that item difficulty was not measured, and other types of validity, such as predictive and concurrent validity were not tested. Therefore, further testing is required to provide more evidence regarding the validity of this tool.

## Conclusion

The success of national development programs largely depends on the health sector’s success in achieving its objectives. The proper use of health care services in different places and among different groups of people is considered a prerequisite for this success [40]. In this case, the clients’ perception of access to health care services is crucial because their perceptions affect when and where they should seek and receive health care services. In this study, the questionnaire on the perception of access to health care services was developed, and its psychometric properties were examined. Therefore, it is expected that related field research will be accelerated with the introduction of this questionnaire. By measuring perceived access to health care services, health policymakers and practitioners may detect and eliminate perceptual and actual obstacles to using health care services.

## Supplementary Information

**Additional file 1.** Technical appendix.

## Data Availability

The datasets analyzed during the current study are available from the corresponding author on reasonable request.

## Footnotes

1 $$ CVR=\frac{\mathrm{ne}-N/2}{N/2} $$

2 impact score (frequency × Importance)
