# Supplementary material for: Psychometric evaluation of the perceived access to health care questionnaire
Source: BMC Health Serv Res. 2021 Jul 2;21:638. doi: 10.1186/s12913-021-06655-2 (PMC8254360; doi:10.1186/s12913-021-06655-2)
Supplement: Supplementary file 1 — Additional file 1. Technical appendix. [file 12913_2021_6655_MOESM1_ESM.docx]

Table A: The items of Questionnaire and their distributions by response

| Latent Variables | Items | | Responses n(%) | | | | | |
| --- | --- | --- | --- | --- | --- | --- | --- | --- |
|  |  |  | absolutely agree | agree | neutral | disagree | absolutely disagree | Total |
| Accessibility | Q1 | The services I need are provided at the health center. | 32(11.6) | 185(67.3) | 24(8.7) | 31(11.3) | 3(1.1) | 275(100) |
|  | Q2 | The distance from the health centers to my house is appropriate. | 88(32.1) | 155(56.6) | 13(4.7) | 14(5.1) | 4(1.5) | 274(100) |
|  | Q3 | The time required to reach the health center is appropriate. | 85(30.8) | 162(58.7) | 9(3.3) | 16(5.8) | 4(1.4) | 276(100) |
|  | Q4 | Getting to and from the health center is easy for me. | 91(33.3) | 159(58.2) | 6(2.2) | 13(4.8) | 4(1.5) | 273(100) |
| Availability | Q5 | The health services (immunization, medical visit, family planning, mother and childcare, injections, etc.) I need are provided at the public health center. | 40(14.7) | 158(57.9) | 33(12.1) | 36(13.2) | 6(2.2) | 273(100) |
|  | Q6 | The facilities of the health center meet the health needs of the clients. | 29(10.6) | 150(54.7) | 38(13.9) | 53(19.3) | 4(1.5) | 274 (100) |
|  | Q7 | Health staff is tailored to the number of clients and their needs. | 30(10.9) | 146(53.3) | 36(13.1) | 54(19.7) | 8(2.9) | 274(100) |
| Acceptability | Q8 | The quality of services provided in the health center is acceptable. | 42(15.5) | 169(62.4) | 28(10.3) | 27(10) | 5(1.8) | 271(100) |
|  | Q9 | The health center staff meets the needs of the clients in various ways, such as being introduced to the community resources. | 57(21.1) | 116(43) | 70(25.9) | 24(8.9) | 3(1.1) | 270(100) |
|  | Q10 | Health workers listen carefully to what I have to say. | 53(20.4) | 172(62.8) | 18(6.6) | 24(8.8) | 4(1.5) | 274(100) |
|  | Q11 | The health workers give me enough time. | 59(21.5) | 157(57.3) | 25(9.1) | 31(11.3) | 2(0.7) | 274(100) |
|  | Q12 | I trust the statements of the treatment team (doctor, nurse, midwife, etc.) about my health and illness | 30(11.1) | 186(68.9) | 28(10.4) | 22(8.1) | 4(1.5) | 270(100) |
|  | Q13 | My request for same sex health care professionals is taken into account. | 32(11.6) | 116(43.3) | 103(38.4) | 15(5.6) | 2(0.7) | 268(100) |
|  | Q14 | I accept screening services such as cervical and colon cancer at this center. | 26(9.7) | 104(39) | 83(31.1) | 41(15.4) | 13(4.9) | 267(100) |
|  | Q15 | The treatment team at the health center is respectful. | 52(19.5) | 189(70.8) | 8(3) | 16(6) | 2(0.7) | 267(100) |
|  | Q16 | Health workers (doctors, nurses, midwives, etc.) are familiar with the culture of clients and communicate with them appropriately. | 40(15) | 162(60.9) | 44(16.5) | 17(6.4) | 3(1.1) | 266(100) |
| Affordability | Q17 | To solve my health problem, I first see a general practitioner. | 43(16) | 139(51.9) | 17(6.3) | 47(17.5) | 22(8.2) | 276(100) |
|  | Q18 | With the guidance of a general practitioner, I use specialized and sub-specialized services. | 46(17.2) | 144(53.9) | 21(7.9) | 33(12.4) | 23(8.6) | 267(100) |
|  | Q19 | Cost is a serious barrier to using health care. | 47(17.7) | 92(34.7) | 15(5.7) | 80(30.2) | 31(11.7) | 265(100) |
| Accommodation | Q20 | It is easy to make an appointment at a health center. | 47(17.6) | 165(61.8) | 13(4.9) | 34(12.7) | 8(3) | 267(100) |
|  | Q21 | The expected time to receive the services I need is appropriate. | 23(8.6) | 169(63.5) | 19(7.1) | 47(17.7) | 8(3) | 266(100) |
|  | Q22 | I can discuss health issues and changes in my condition over the phone (in person) with the treatment team (doctor, nurse, midwife, etc.). | 12(4.4) | 36(13.3) | 71(26.3) | 81(30) | 70(25.9) | 270(100) |
|  | Q23 | The working hours of the public health center are suitable for receiving services from these centers. | 17(6.4) | 162(60.7) | 24(9) | 48(18) | 16(6) | 267(100) |
|  | Q24 | The physical space of the health center is suitable for receiving services. | 33(12.3) | 191(71.3) | 23(8.6) | 16(6) | 5(1.9) | 268(100) |
|  | Q25 | Access to some facilities such as wheelchairs, walkers, etc. is provided in the health center. | 10(3.8) | 39(14.7) | 123(46.2) | 61(22.9) | 33(12.4) | 266(100) |
| Awareness | Q26 | The educations that are given to me are such that I understand them. | 34(12.7) | 183(68.5) | 33(12.4) | 14(5.2) | 3(1.1) | 267(100) |
|  | Q27 | The information I need is expressed in simple language without the use of specialized words. | 35(13.3) | 188(71.2) | 28(10.6) | 11(4.2) | 2(0.8) | 264(100) |
|  | Q28 | Communication of health workers (doctor, nurse, midwife, etc.) with clients is appropriate. | 35(13) | 206(76.6) | 16(5.9) | 11(4.1) | 1(0.4) | 269(100) |
|  | Q29 | Health workers try to make sure I fully understand the health information provided. | 28(10.5) | 163(61) | 35(13.1) | 39(14.6) | 2(0.7) | 267(100) |
|  | Q30 | My living conditions are taken into accounts, such as marital status, ability to pay, and cultural differences. | 17(6.3) | 63(23.5) | 86(32.1) | 89(33.2) | 13(4.9) | 268(100) |
